# Correlation of *In Vivo* and *Ex Vivo* ADC and T2 of *In Situ* and Invasive Murine Mammary Cancers

**DOI:** 10.1371/journal.pone.0129212

**Published:** 2015-07-24

**Authors:** Xiaobing Fan, Kay Macleod, Devkumar Mustafi, Suzanne D. Conzen, Erica Markiewicz, Marta Zamora, James Vosicky, Jeffrey Mueller, Gregory S. Karczmar

**Affiliations:** 1 Department of Radiology, The University of Chicago, 5841 S. Maryland Avenue, Chicago, IL, 60637, United States of America; 2 Ben May Department for Cancer Research, The University of Chicago, 929 East 57th Street, Chicago, IL, 60637, United States of America; 3 Medicine, Hematology/Oncology, The University of Chicago, 5841 S. Maryland Avenue, Chicago, IL, 60637, United States of America; 4 Department of Pathology, The University of Chicago, 5841 S. Maryland Avenue, Chicago, IL, 60637, United States of America; Northwestern University Feinberg School of Medicine, UNITED STATES

## Abstract

*Ex vivo* MRI may aid in the evaluation of surgical specimens, and provide valuable information regarding the micro-anatomy of mammary/breast cancer. The use of *ex vivo* MRI to study mouse mammary cancer would be enhanced if there is a strong correlation between parameters derived from *in vivo* and *ex vivo* scans. Here, we report the correlation between apparent diffusion coefficient (ADC) and T_2_ values measured *in vivo* and *ex vivo* in mouse mammary glands with *in situ* cancers (mammary intraepithelial neoplasia (MIN)) and invasive cancers (those which spread outside the ducts into surrounding tissue). MRI experiments were performed on the Polyoma middle T oncoprotein breast cancer mouse model (n = 15) in a 9.4T scanner. For *in vivo* experiments, T_2_-weighted (T2W) images were acquired to identify abnormal regions, then ADC and T_2_ values were measured for nine selected slices. For *ex vivo* experiments, a midline incision was made along the spine, and then skin, glands, and tumors were gently peeled from the body. Tissue was fixed in formalin, placed around a mouse-sized sponge, and sutured together mimicking the geometry of the gland when attached to the mouse. The same pulse sequences used for *in vivo* experiments were repeated for *ex vivo* scans at room temperature. Regions of interest were manually traced on T2W images defining features that could be identified on *in vivo* and *ex vivo* images. The results demonstrate a strong positive correlations between *in vivo* and *ex vivo* invasive cancers for ADC (r = 0.89, p <0.0001) and T_2_ (r = 0.89, p <0.0001) values; and weak to moderate positive correlations between *in vivo* and *ex vivo in situ* cancers for ADC (r = 0.61, p <0.0001) and T_2_ (r = 0.79, p <0.0001) values. The average *ex vivo* ADC value was about 54% of the *in vivo* value; and the average *ex vivo* T_2_ was similar to the *in vivo* value for cancers. Although motion, fixation, and temperature differences affect ADC and T_2_, these results show a reliable relationship between ADC and T_2_ *in vivo* and *ex vivo*. As a result *ex vivo* images can provide valuable information with clinical and research applications.

## Introduction

*Ex vivo* imaging of human breast cancer and murine mammary cancer has both clinical and research applications. Magnetic resonance imaging (MRI) shows lesion anatomy and margins accurately *in vivo* [1,2]. If the contrast in *in vivo* and *ex vivo* images is similar, this suggests MRI can aid intra-operative assessments of tumor margins in lumpectomy specimens. Intra-operative radiographs are currently used to identify tumor margins and this decreases re-excision rates [3,4], but X-ray imaging does not provide optimal soft tissue contrast. MRI has potential to improve intra-operative imaging by providing high resolution three-dimensional images with excellent soft tissue contrast. In addition, *ex vivo* images can serve as a ‘bridge’ between *in vivo* images and fixed tissue, to aid co-registration of MRI and histology. Finally, high resolution *ex vivo* imaging of breast/mammary cancers could provide new information concerning three-dimensional structure, and this may be particularly useful for studies of *in situ* cancers [5].

All of these potential applications of *ex vivo* imaging would be facilitated if there is a strong correlation between MRI parameters measured *in vivo* and *ex vivo*. This correlation would suggest that contrast in *in vivo* and *ex vivo* images is similar, and therefore, *ex vivo* images provide useful information concerning the structure and location of breast/mammary cancers.

Apparent diffusion coefficient (ADC) and T_2_ are important sources of contrast in breast imaging that do not require contrast media injection. Therefore, they are particularly relevant for *ex vivo* imaging. Previous studies have compared contrast in *in vivo* and *ex vivo* diffusion and T_2_-weighted images. For example, Kim et al. [6] demonstrated that ADC values of the carotid plaque components *in vivo* were consistent with values obtained from *ex vivo* endarterectomy specimens. Sun et al. [7] compared *in vivo* and *ex vivo* ADCs of hepatic tumors, and showed that ADCs were significantly smaller in postmortem tumor and liver compared to *in vivo* values. Takano et al. [8] showed that T_2_ for the spinal cords of mice was significantly higher *in vivo* than *ex vivo*.

In this study we evaluate whether there is a correlation between ADC and T_2_ *in vivo* and ADC and T_2_ of formalin-fixed mammary cancers in polyoma middle T (PyMT) transgenic mice–a widely used model of human breast cancer [9]. In PyMT mice, four distinct identifiable stages of tumor progression from premalignant to malignant stages are observed. These include hyperplasia, adenoma/mammary intraepithelial neoplasia (MIN), and early- and late-stage carcinoma. These stages are comparable to human breast diseases classified as benign lesions, *in situ* proliferative lesions, and invasive carcinomas. Here, we refer to ‘adenoma/mammary intraepithelial neoplasia (MIN)’ as ‘*in situ* cancer’ and ‘early and late carcinoma’ as ‘invasive cancer’. A novel method for comparing *in vivo* and *ex vivo* images was developed to investigate this relationship. Anatomic and functional MRI studies of this model have the potential to provide important new information regarding breast/mammary cancer initiation and progression [5,10,11]. In particular, *ex vivo* MRI allows evaluation of mouse mammary glands at very high spatial resolution. However, formalin fixation changes tissue microstructure [12] and this is expected to affect ADC and T_2_. An understanding of the relationship between ADC and T_2_ *in vivo* versus *ex vivo* will aid interpretation of MRI studies of mammary/breast cancer anatomy *ex vivo*.

## Materials and Methods

### Animals

A spontaneously metastasizing transgenic model of breast cancer was used in this research. Cancer is induced by the polyoma middle T antigen (PyMT) driven by the murine mammary tumor virus promoter (MMTV). BNIP3 is a major factor in promotion of mitochondrial autophagy [13]. The PyMT mice with and without BNIP3 suppressed are referred to as knockout and wild type in this study, respectively. Both types of mice developed mammary cancers at ~10–11 weeks. MMTV-PyMT mice were purchased from JAX (strain # 2374) (JAX Mice, Clinical & Research Services, Bar Harbor, Maine USA) on an FVB/N genetic background [14]. All mice were handled and euthanized in accordance with protocols approved by the University of Chicago's Institutional Animal Care and Use Committee (IACUC) (Protocol Number: 71155). Humane endpoints were used, consistent with the approved IACUC protocol. Mice were euthanized when tumor volume exceeding 2 cm^3^ or tumors became ulcerated, or if there was weight loss of more than 20% of body weight.

A total of 15 PyMT mice (10–11 weeks old), including 5 knockout and 10 wild type mice, were used for *in vivo* and *ex vivo* imaging experiments. Invasive mammary cancers developed in all of these mice. However, the knockout and wild type mice have different tumor growth rates and different times to metastasis to lung. Therefore, use of these two different mouse models allowed us to study the correlation between *in vivo* and *ex vivo* MRI parameters in cancers with a larger range of sizes and stages.

Animals were anesthetized before imaging experiments, and anesthesia was maintained during imaging at 1.5% isoflurane. The temperature, heart rate and respiration rate were monitored with an optical detection system from SA Instruments (Stony Brook, NY, USA), developed for use in small animal MRI. The respiration rate was maintained at ~55 breaths per minute and used to obtain gated images.

### *In vivo* MRI experiments

MRI experiments were performed on a 9.4 Tesla Bruker (Billerica, MA, USA) small animal scanner with 11.6 cm inner diameter, actively shielded gradient coils (maximum constant gradient strength for all axes: 230 mT/m). Whole-body scanning was performed to study all of the mammary glands. Mice were taped into a plastic semi-circular holder and placed inside a volume RF quadrature coil (Bruker BioSpin MRI GmbH Quad coil, OD/ID  =  59/35 mm, length  =  38 mm). For *in vivo* experiments, multi-slice RARE (Rapid Acquisition with Relaxation Enhancement) spin echo T_2_-weighted (T2W) images with fat suppression and getting (TR/TE_effective_ = 4000/26 ms, field of view (FOV) = 25.6 mm, matrix size = 256^2^, slice thickness = 0.5 mm, NEX = 2, RARE factor = 4) were acquired from upper and lower mammary glands separately to identify abnormal regions. For lower glands only, diffusion weighted images (DWI) were acquired using a spin echo for signal acquisition without gating (TR/TE = 4000/26 ms, b-value = 0, 500, 1000, and 1500 s/mm^2^, FOV = 32 mm, matrix size = 128^2^, slice thickness = 1.0 mm, NEX = 1) for nine slices selected based on the T2W images. The T_2_ values were measured using a multi-slice-multi-echo sequence without gating (TR = 4000 ms, number of echoes = 24, 1^st^ TE = 12.5 ms, increment of TE = 12.5 ms) at the same nine slices as DWI. Four mice died before the T_2_ measurements were completed.

### *Ex vivo* MRI experiments

For *ex vivo* experiments, the skin and glands were taken by carefully excising the skin from the mouse. A midline incision along the back spine was made from the tail to the head; and then the skin, glands, and tumors were gently peeled from the body muscle so that the hide remained intact. The tissue was fixed in formalin for a minimum of seven days, then washed in phosphate buffered saline for five days to remove the formalin, because formalin containing tissue has a significantly shortened T_2_. Subsequently the fixed skin was placed around a mouse-sized sponge and sutured together back along the midline to mimic the geometry of the gland when attached to the mouse *in vivo*. This greatly facilitated reliable identification of corresponding features on *in* vivo and *ex vivo* images. This skin was then placed in a larger tube filled with fomblin and sealed before being placed into the resonator. The same pulse sequences (without gating) used for *in vivo* experiments were repeated for *ex vivo* experiments at room temperature (22°C).

### Image analysis

The data were processed and analyzed using software written in IDL (ITT Visual Information Solutions, Inc., Boulder, CO, USA). For ADC and T_2_ measurements, the k-space data were zero-padded prior to Fourier transform so that the final image size was four times larger than the original image. This greatly facilitated tracing regions of interest (ROI) on both *in vivo* and *ex vivo* MRI. Pixel-by-pixel analysis was performed to obtain ADC maps and T_2_ maps. The ADC in each pixel was calculated by fitting the raw data using the following equation:

$$S_{b}=S_{SE}\;\text{exp}(-b\cdot D)$$ (1)

where S_b_ is the attenuated spin-echo signal and S_SE_ is the maximum spin-echo signal without diffusion attenuation. T_2_ was calculated by fitting the raw data with the equation:

$$S_{TE}=S_{0}\;\text{exp}(-TE/T_{2})$$ (2)

where S_0_ is the extrapolated signal at TE = 0 and the S_TE_ is signal measured at each TE.

ROIs were manually traced on T2W images to define features that could be visually and unambiguously identified on both *in vivo* and *ex vivo* images. The ROI boundaries were traced within the edges of each feature to minimize partial volume effects. The same ROIs were used to obtain the ADC and T_2_ values. The ROIs were identified based on consensus between researchers (XF and EM) with 15 years and 8 years of experience with imaging mouse mammary glands. Because the *ex vivo* mammary glands were placed in approximately the same configuration as the *in vivo* glands (as described above) and because the features of interest were relatively sparse, corresponding features on *in vivo* and *ex vivo* glands could be identified unambiguously.

A total of 10–15 pairs of ROIs of similar sizes were traced for each mouse. They included lymph nodes, *in situ* cancers, and invasive cancers, identified based on previous work. Previous studies correlated features identified on MRI with histology and established that small scattered foci (from one to three hundred microns in diameter) with increased intensity on T2W images, and with elongated regions of high intensity (resembling individual ducts), are almost always *in situ* cancers [11]. Invasive cancers were identified as solid masses greater than ~0.5 mm in diameter, with intensity higher than muscle on T2W images. Lymph nodes were identified based on their location, oval shape, and intensity close to that of muscle on T2W images.

All ROIs for lymph nodes were pooled together for comparison of *in vivo* and *ex vivo* ADC and T2 values. Similarly ROIs for *in situ* cancers were pooled, and ROIs for invasive cancers were pooled. For each group of ROI’s, paired t-tests were used to compare *in vivo* and *ex vivo* ADC and T_2_ values. One-way ANOVA and Tukey's HSD (honestly significant difference) tests were performed to determine whether ADC (T_2_) values for lymph nodes, *in situ* cancers, and invasive cancers were significantly different on *in vivo* scans and the same tests were performed for *ex vivo* scans. The Pearson correlation test was performed to examine whether there is a linear relationship between *in vivo* and *ex vivo* ADC (T_2_) values. A p-value less than 0.05 was considered significant.

## Results

**Fig 1:**
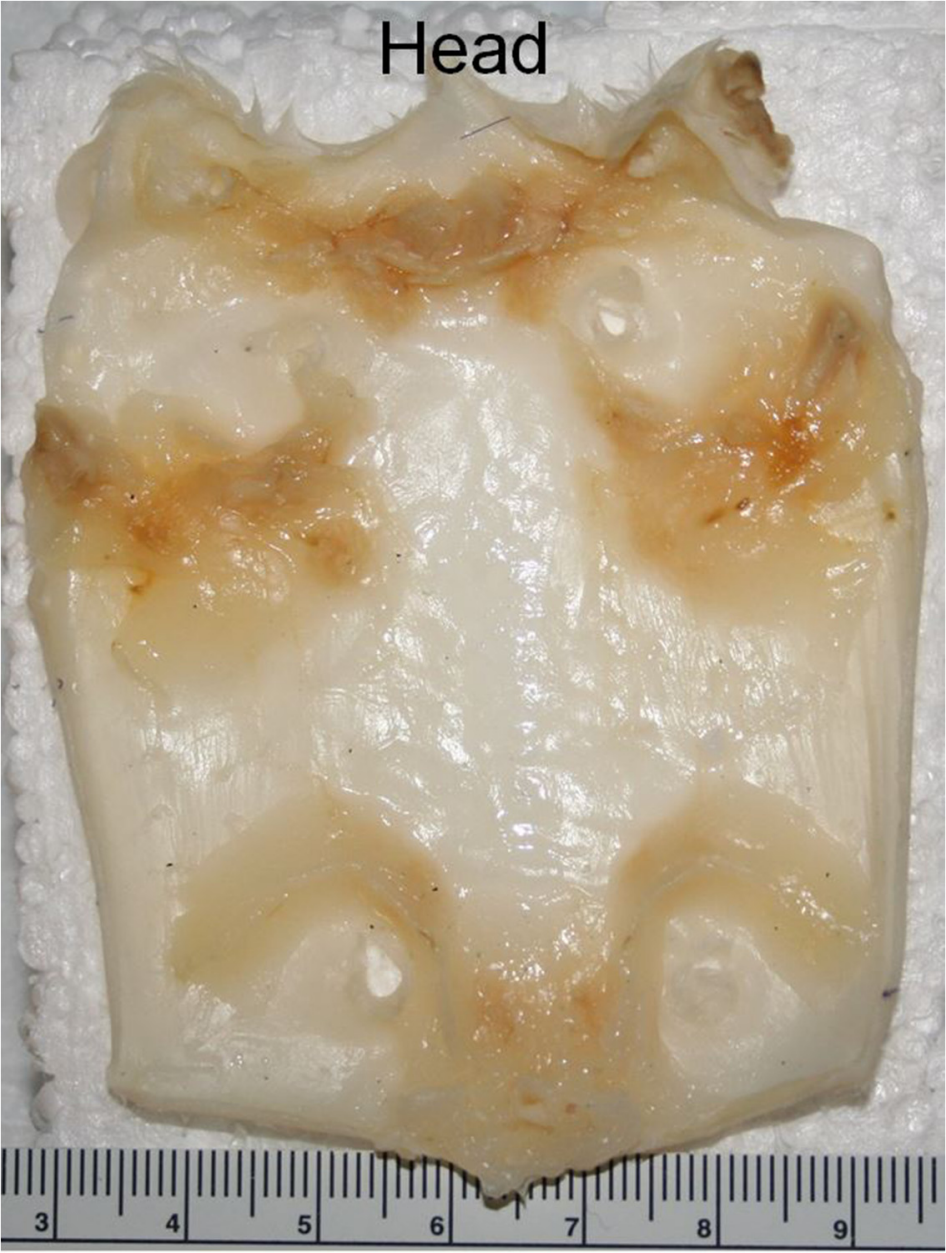
Photograph of excised skin from a mouse after treatment with formaldehyde before preparation for *ex vivo* imaging. The scale of the ruler is in millimeters.

**Fig 2:**
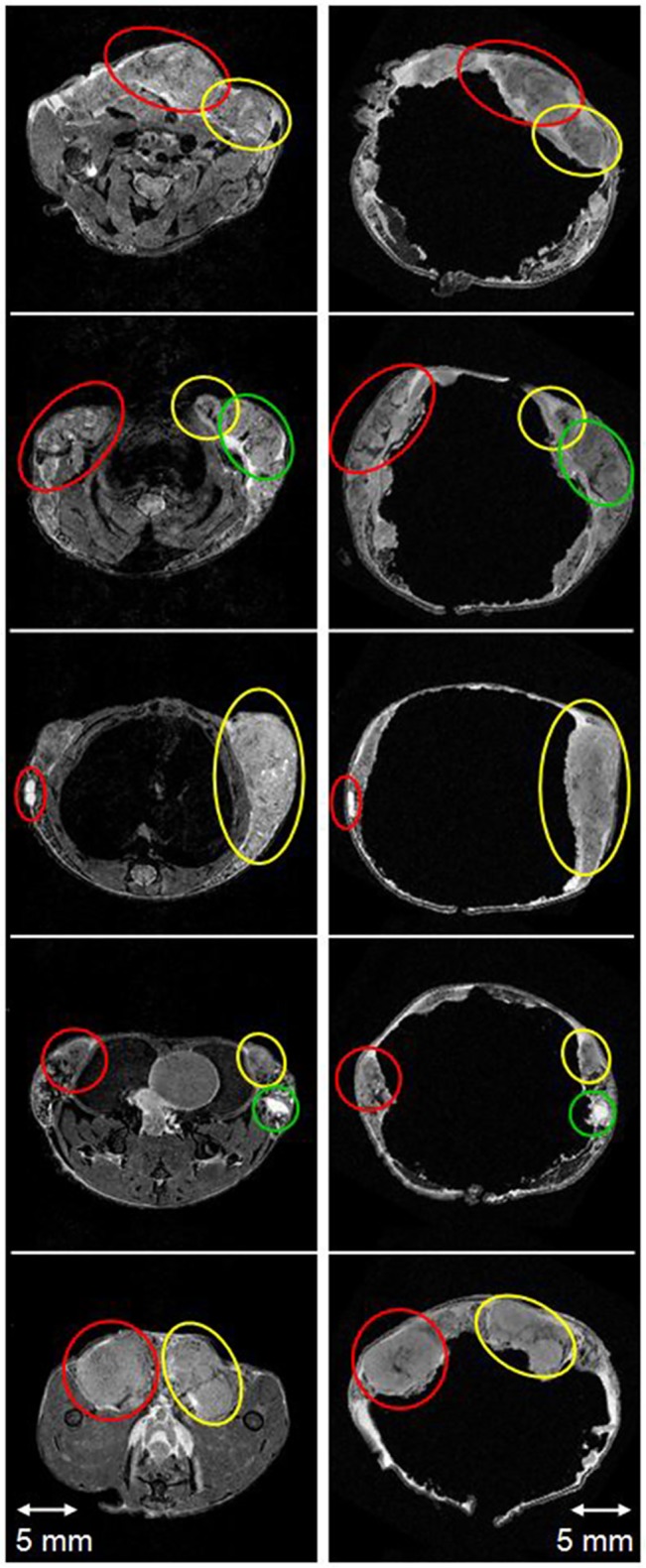
T2W *in vivo* image (left panel) matched with the corresponding *ex vivo* image (right panel) showing a mouse mammary gland from head to tail (top to bottom)–near the neck, heart, liver, below the kidney, and near the legs, respectively. Matching features (all invasive cancers) in the *in vivo* and *ex vivo* images, identified by visual inspection, were circled with the same color. The displayed image FOV is 25.6 × 25.6 mm.

Immediately after the *in vivo* MRI experiments, the mouse skin and glands were carefully removed from the body. Fig 1 shows an example of the excised skin after fixation and ready for *ex vivo* imaging. During the fixing process, the skin shrinks or stretches slightly compared to *in vivo* skin. The mouse skin was then sutured together around a mouse-sized sponge for *ex vivo* MRI. Fig 2 compares *in vivo* (left panel) and *ex vivo* (right panel) T2W images from a single mouse, three slices from the top glands and two slices from bottom glands. Gross features, all invasive cancers, indicated by circles of the same color, are well matched, despite the change in *ex vivo* lesion shape and size. Because the features selected for analysis are sparse, the corresponding features on *in vivo* and *ex vivo* images can be identified unambiguously.

**Fig 3:**
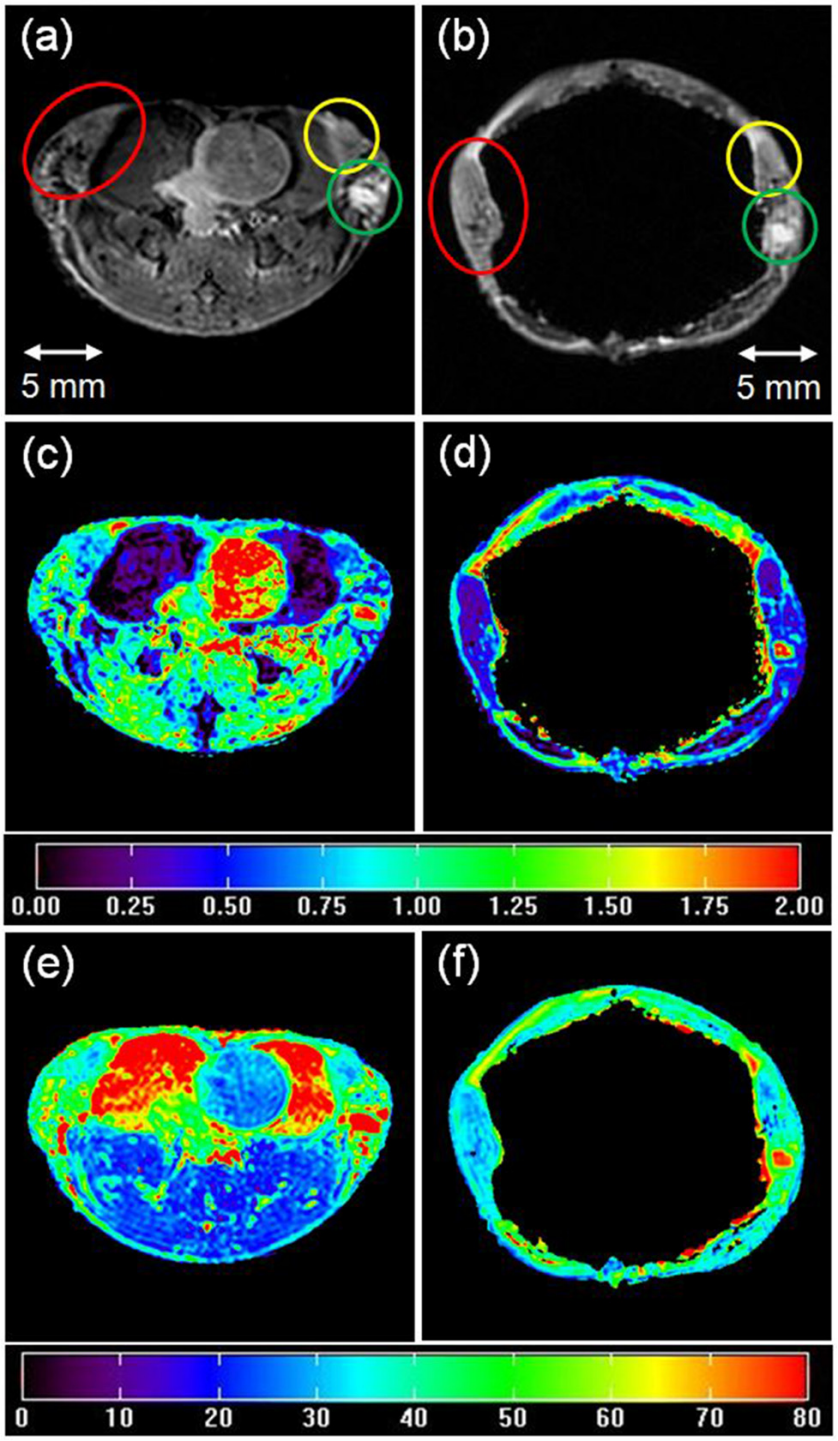
Matched invasive cancers (circled by the same color) from *in vivo* images (left panel) and *ex vivo* images (right panel) of a mouse mammary gland. (a, b) T2W images; (c, d) ADC maps (×10^−3^ mm^2^/s); (e, f) T_2_ maps (ms). The displayed image FOV is 25.6 × 25.6 mm.

**Fig 4:**
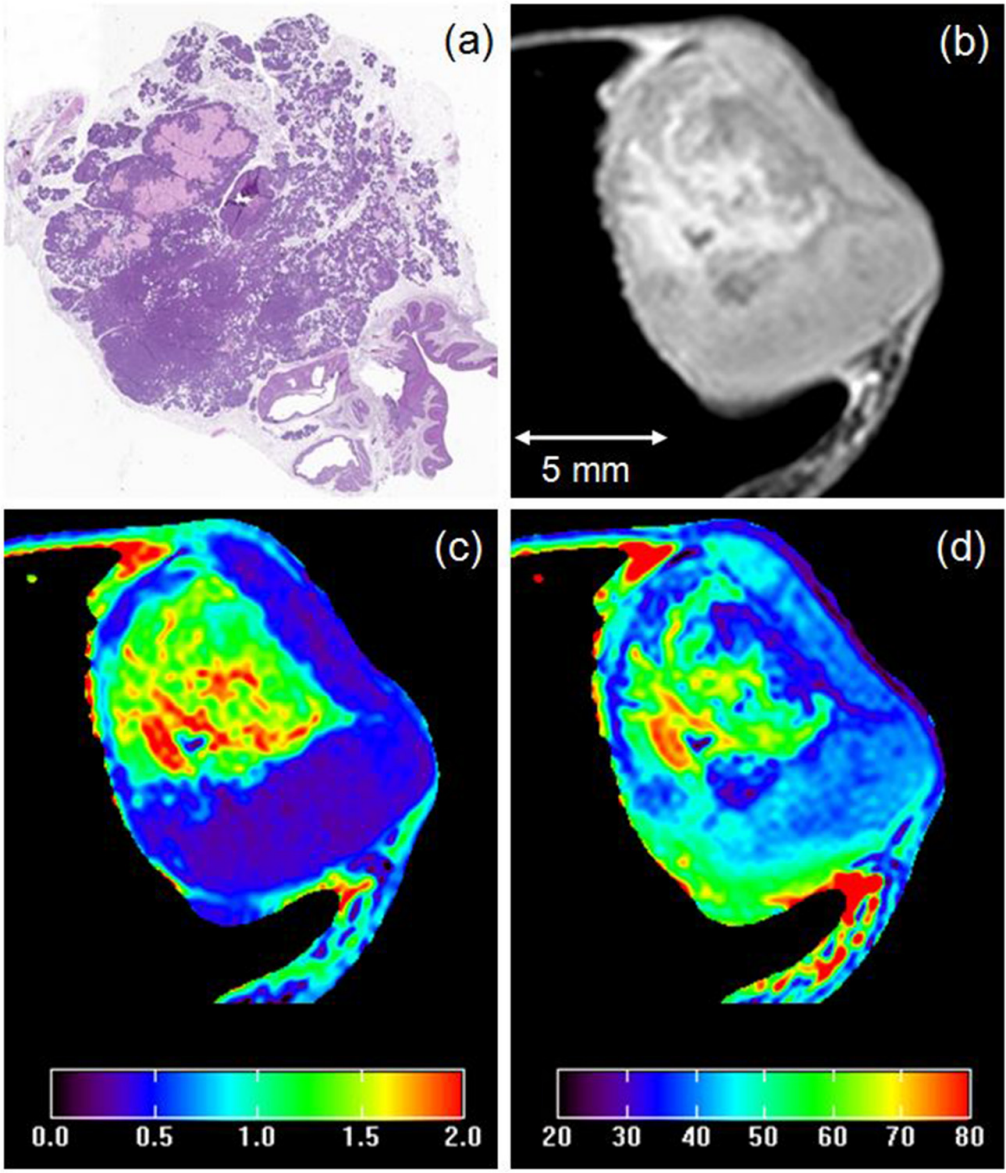
(a) H&E stained slice through an invasive cancer, (b) *ex vivo* T2W image, (c) corresponding ADC map (×10^−3^ mm^2^/s), and (d) T_2_ map (ms). The displayed image FOV is 15.0 × 15.0 mm.

The ADC and T_2_ maps were generated using Eqs 1 and 2. Mono-exponential functions (Eqs 1 and 2) provided excellent fits to ADC and T_2_ data from mammary glands, with average goodness-of-fit values of 0.96 and 0.99, respectively. The ADC and T_2_ maps produced by these fits are shown in Fig 3 for typical slices *in vivo* and *ex vivo*. ADC and T_2_ values varied widely across the tumor; the ADC was especially heterogeneous. For example, Fig 4 shows (a) an invasive cancer on an H&E stained slice, (b) an *ex vivo* T2W image, (c) the corresponding ADC map, and (d) the T_2_ map. For the cross section of the tumor shown in Fig 4, the average (± standard deviation) *ex vivo* ADC was 0.87 ± 0.53 ×10^−3^ mm^2^/s; and the average T_2_ (± standard deviation) was 45.7 ± 10 ms.

**Fig 5:**
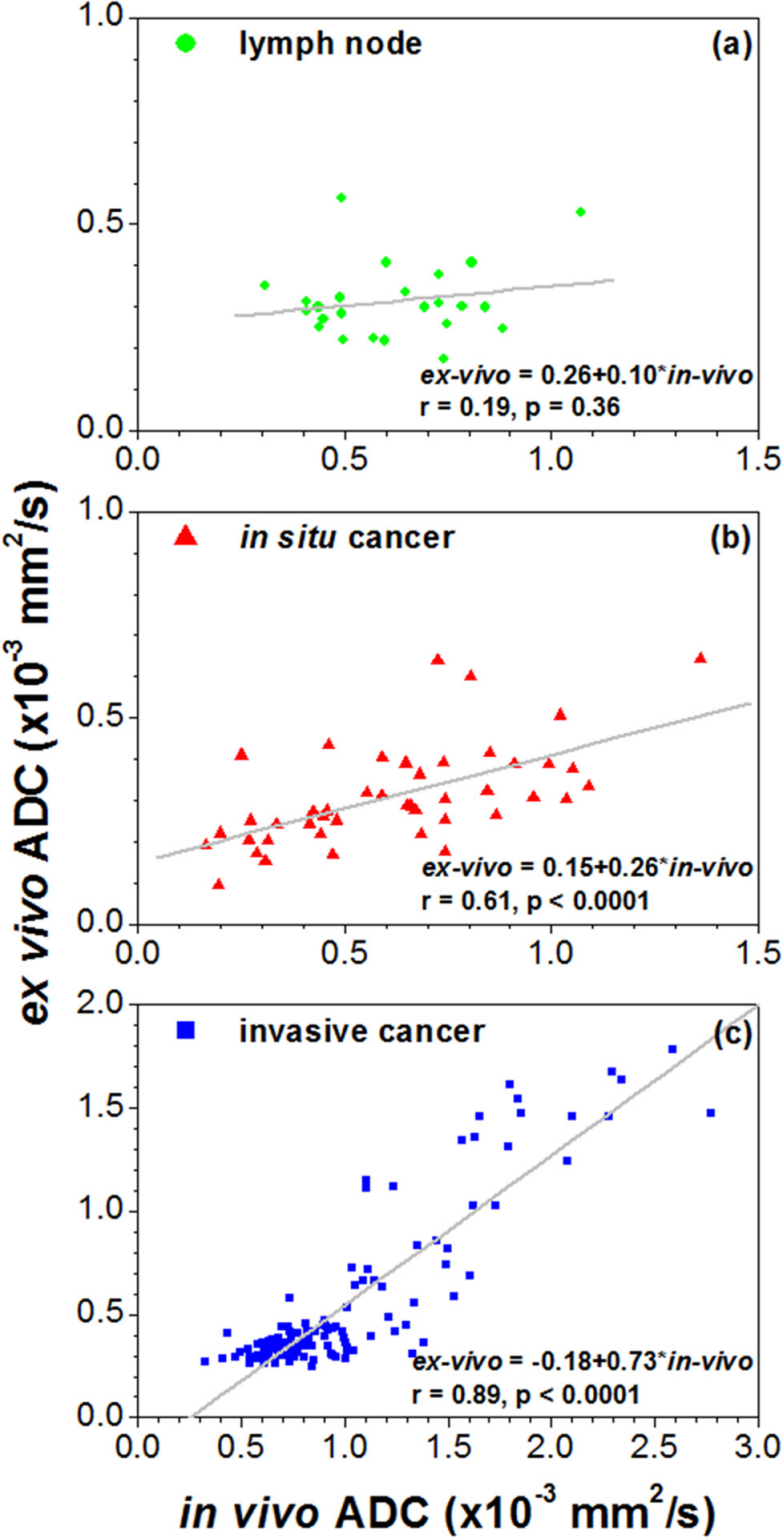
Plots of *in vivo* versus *ex vivo* average ADC values over ROIs for all 15 mice. (a) lymph nodes, (b) *in situ* cancers, and (c) invasive cancers. The gray line is the linear fit through the points. The linear relationship between *in vivo* and *ex vivo* of ADC, the correlation coefficient (r) and p value are given on the plot.

For visually matched features in *in vivo* and *ex vivo* mammary glands, the average ADC values were calculated over the manually traced ROIs in lymph nodes, *in situ* cancers, and invasive cancers (a total of 187 *in vivo* and *ex vivo* pairs of ROI’s from 15 mice, Table 1). Fig 5 shows plots of the *in vivo* vs. *ex vivo* ADC values averaged over ROIs for 15 different mice, including data from lymph nodes, *in situ* cancers, and invasive cancers. There is a strong positive correlation (r = 0.89, p < 0.0001) between *in vivo* and *ex vivo* ADCs for invasive cancers, and a weaker but statistically significant positive correlation between *in vivo* and *ex vivo* ADCs for *in situ* cancers. There is no correlation (r = 0.19, p = 0.36) between *in vivo* and *ex vivo* ADCs for lymph nodes. Considering all three tissue types examined, paired t-test showed that *in vivo* ADC values were significantly larger (p < 0.0001) than *ex vivo* values. The average *ex vivo* ADC was about 54% of the *in vivo* value (Table 2). One-way ANOVA and Tukey's HSD showed that the *in vivo* and *ex vivo* ADC values for invasive cancers were significantly larger (p < 0.001) than for lymph nodes and *in situ* cancers.

**Table 1:** Number of ROIs for lymph nodes, *in situ* cancers, and invasive cancers found in each mouse (named A to O) that matched between *in vivo* and *ex vivo* MRI experiments.

Mouse	lymph nodes	*in situ* cancers	invasive cancers
**knockout**			
A	1	3	10
B	1	2	11
C	2	4	4
D	1	3	6
E	2	3	5
**wild type**			
F	0	1	13
G	2	6	6
H	1	1	13
I	1	3	9
J	2	5	7
K	2	1	8
L	3	4	4
M	2	2	7
N	2	1	8
O	2	5	8
**Total # ROIs**	24	44	119

Note that four mice (A, B, F and G) died before T_2_ measurements could be completed.

**Table 2:** The average *in vivo* and *ex vivo* ADC values (mean ± standard deviation) for lymph nodes, *in situ* cancers, and invasive cancers.

	lymph nodes (n = 24)	*in situ* cancers (n = 44)	invasive cancers (n = 119)
***in vivo* ADC** (×10^−3^ mm^2^/s)	0.62±0.19	0.62±0.28	1.02±0.48
***ex vivo* ADC** (×10^−3^ mm^2^/s)	0.31±0.09	0.31±0.12	0.56±0.39

‘n’ is the number of pairs of *in vivo* and *ex vivo* ROIs identified in each category.

**Fig 6:**
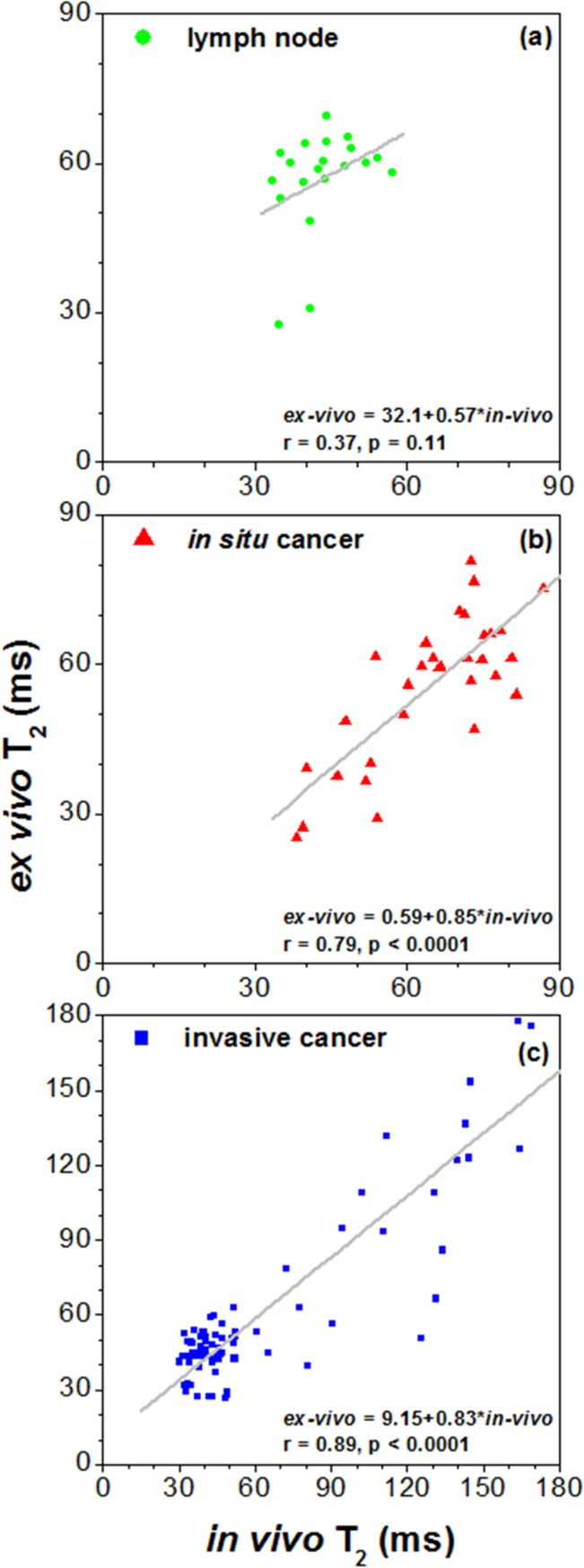
Plots of *in vivo* versus *ex vivo* average T_2_ values over ROIs from 11 mice. (a) lymph nodes, (b) *in situ* cancers, and (c) invasive cancers. The gray line is the linear fit through the points. The linear relationship between *in vivo* and *ex vivo* T_2_s, the correlation coefficient (r) and p value are given on the plot.

T_2_ values were calculated in mammary gland ROIs from 11 of the mice (131 different ROIs, 4 mice died before measurements could be completed, Table 1). Fig 6 shows the plots of *in vivo* vs. *ex vivo* T_2_ values, averaged over ROIs from lymph nodes, *in situ* cancers, and invasive cancers. There is a strong positive correlation (r = 0.89, p < 0.0001) between *in vivo* and *ex vivo* T_2_s for invasive cancers, and a moderate but statistically significant positive correlation (r = 0.79, p < 0.0001) between *in vivo* and *ex vivo* T_2_s for *in situ* cancers. There is no correlation (r = 0.37, p = 0.11) between *in vivo* and *ex vivo* T_2_s for lymph nodes. Paired t-test showed that the *in vivo* T_2_ values were significantly higher (p < 0.001) than *ex vivo* values for *in situ* cancers, but significantly lower for lymph nodes (p < 0.001). The average *in vivo* T_2_ for invasive cancers was about the same as *ex vivo* T_2_ (p > 0.05) (Table 3). One-way ANOVA and Tukey's HSD showed that the *in vivo* T_2_ values for lymph nodes were significantly lower (p < 0.001) than *in vivo* T_2_ values for *in situ* cancers and invasive cancers. However, *ex vivo* T_2_ values were not significantly different between lymph nodes, *in situ* cancers, and invasive cancers.

**Table 3:** The average *in vivo* and *ex vivo* T_2_ values (mean ± standard deviation) for lymph nodes, *in situ* cancers, and invasive cancers.

	lymph nodes (n = 20)	*in situ* cancers (n = 32)	invasive cancers (n = 79)
***in vivo* T**_**2**_ (ms)	43.1±6.70	64.5±13.1	62.9±40.7
***ex vivo* T**_**2**_ (ms)	56.7±10.4	55.7±14.2	61.0±37.6

‘n’ is the number of pairs of *in vivo* and *ex vivo* ROIs identified in each category.

## Discussion

These results demonstrate strong positive correlations between *in vivo* and *ex vivo* mouse mammary invasive cancers for ADC (r = 0.89, p < 0.0001) and T_2_ (r = 0.89, p < 0.0001) values; and weak to moderate, but statistically significant positive correlations between *in vivo* and *ex vivo* mouse mammary *in situ* cancers for ADC (r = 0.61, p < 0.0001) and T_2_ (r = 0.79, p < 0.0001) values. The average *ex vivo* ADC was about 0.54 times the *in vivo* value. The lower *ex vivo* ADC is consistent with previously published reports [15]. The ADC is known to increase with temperature at a rate of 2.4%/°C [16]. If this correction is applied to the data, the *ex vivo* ADC increases from 54% of the *in vivo* value to 73% of the *in vivo* value. The remaining difference between *ex vivo* and *in vivo* ADCs could be due to structural changes caused by formalin fixation, the effect of perfusion, convection or motion of the mouse *in vivo*, changes in membrane permeability, or the absence of energy dependent active-water transport via ion pumps in *ex vivo* tissue [17]. Although the average *ex vivo* T_2_ was about the same as the *in vivo* value for invasive cancers, the average *ex vivo* T_2_ was about 9 ms shorter (p < 0.001) and 14 ms longer (p < 0.001) than *in vivo* T_2_s for *in situ* cancers and lymph nodes, respectively. *In vivo* T_2_’s differentiated between cancers and lymph nodes, but *ex vivo* T_2_’s did not. This could be due to the effects of formalin fixation and/or to residual deoxygenated blood.

Because of small number of knockout mice used in this study, we could not accurately determine whether there was a difference in cancers ADCs and T_2_s between knockout and wild type mice. This important issue will be addressed in future research. Due to a lack of landmarks and the stretching or shrinking of skin causing deformation in the *ex vivo* images, comparisons on a pixel-by-pixel basis are not possible. Nevertheless, small, distinct features, such as lymph nodes and small lesions, were reliably compared on *in vivo* and *ex vivo* images. To our knowledge, this is the first report of correlation between *in vivo* and *ex vivo* MRI of mouse mammary glands. Because the *ex vivo* images were placed on a circular form, features found on *in vivo* slices were reliably identified on *ex vivo* slices.

The ADC and T_2_ values calculated from both *in vivo* and *ex vivo* data were consistent with previously published values [8,18,19]. Park et al. [20] using a maximum b-value of 1000 s/mm^2^, found that the mean ADC of the invasive ductal carcinoma was 0.89 ± 0.18 ×10^−3^ mm^2^/s and the mean ADC of ductal carcinoma *in situ* (DCIS) was 1.17 ± 0.18 ×10^−3^ mm^2^/s. Both of these ADC’s were significantly lower than those of the benign lesions 1.41 ± 0.56 ×10^−3^ mm^2^/s. Other studies using smaller b-values reported larger ADC’s [21]. The mean ADCs reported here for invasive and *in situ* cancers in mouse mammary glands are close to but smaller than the ADC’s reported by Park et al. Invasive murine cancers were very heterogeneous, with a large range of ADCs, as shown in Fig 4. The range of ADC’s in *in situ* cancers was much smaller–as shown in Fig 5, suggesting that *in situ* cancers may be less heterogeneous on DWI than invasive cancers.

In the present study, as well as in DWI of patients, diffusion measurements for small *in situ* cancers suffer from partial volume effects that produce errors in ADC measurements. Here we used the same resolution for *in vivo* and *ex vivo* measurements, and this may have resulted in partial volume effects and somewhat lower ADC’s measured for *in situ* cancers compared to invasive cancers. This may explain the differences between both *in vivo* and *ex vivo* ADC’s of *in situ* and invasive cancers. However, the excellent quality of the *ex vivo* images suggests that in the future much higher resolution *ex vivo* images could be acquired so that the ADC of *in situ* cancers relative to invasive cancers can be more accurately determined. Because of the excellent correlation between *in vivo* and *ex vivo* ADC’s reported here, *ex vivo* ADC measurements would provide useful information concerning the physical characteristics of *in situ* cancers.

In the present study, we used 4 b-values up to a maximum of 1500 s/mm^2^. The simple model used for data analysis did not take perfusion into account. Despite the fact that we did not correct for the potential effect of perfusion on *in vivo* data–the correlation between *in vivo* and *ex vivo* results was very strong. In future work–a larger number of b-values, and more complex models could be used to further improve the correlation.

Although the absolute values differ, the strong correlation between *in vivo* and *ex vivo* images suggests that contrast in *in vivo* and *ex vivo* ADC and T_2_ images is similar, and that morphology of breast/mammary cancers on MRI *ex vivo* is relevant to *in vivo* images. As a result, it is likely that motion-free, high resolution *ex vivo* images can provide new and useful information regarding tumor structure that is not available from *in vivo* images; this may be particularly important for small *in situ* cancers. *Ex vivo* imaging could be used as a starting point for optimizing methods and protocols for ADC and T_2_ imaging that most effectively separate lymph nodes, *in situ* cancers and invasive cancers *in vivo*. In addition, *ex vivo* imaging could serve as an aid to pathologists to identify tumor margins and improve the sensitivity, specificity, and speed with which surgical specimens can be evaluated.
